# Effect of COVID-19 on the Number of CT-scans and MRI Services of Public Hospitals in Iran: An Interrupted Time Series Analysis

**DOI:** 10.4314/ejhs.v31i6.5

**Published:** 2021-11

**Authors:** Mohammad Heydarian, Masoud Behzadifar, Christos V Chalitsios, Mohammad Keshvari, Roodabeh Omidifar, Mahboubeh Khaton Ghanbari, Hasan Abolghasem Gorji, Jude Dzevela Kong, Jianhong Wu, Nicola Luigi Bragazzi

**Affiliations:** 1 Department of Radiology, Faculty of Medicine, Lorestan University of Medical Sciences, Khorramabad, Iran; 2 Social Determinants of Health Research Center, Lorestan University of Medical Sciences, Khorramabad, Iran; 3 Division of Respiratory Medicine, Clinical Science Building, School of Medicine, University of Nottingham, Nottingham NG5 1PB, UK; 4 Division of Epidemiology and Public Health, Clinical Science Building, School of Medicine, University of Nottingham, Nottingham NG5 1PB, UK; 5 Vice Chancellor Office, Lorestan University of Medical Sciences, Khorramabad, Iran; 6 Health Management Research Institute, Iran University of Medical Sciences, Tehran, Iran; 7 Laboratory for Industrial and Applied Mathematics (LIAM), Department of Mathematics and Statistics, York University, Toronto, Canada; 8 School of Public Health, Department of Health Sciences (DISSAL), University of Genoa, Genoa, Italy

**Keywords:** COVID-19, CT-Scans, MRI, Interrupted time series, Iran, Health services

## Abstract

**Background:**

In February 2020, the Ministry of Health and Medical Education in Iran announced the first case of COVID-19. The aim of this study was to investigate the impact of COVID-19 on the number of CT-Scans and MRI services in public hospitals in western Iran.

**Methods:**

We collected CT-scans and MRI services data from 18 public hospitals via Vice-Chancellor Office, Lorestan University of Medical Sciences from January 2017 to February 2021. Interrupted time series analysis (ITSA) was conducted to assess the impact of COVID-19 on CT-Scans and MRI services. More specifically, ITSA was conducted using ordinary least squares regression with the number of CT-Scans and MRI services per 1,000 registered persons per month as dependent variable.

**Results:**

At the beginning of the observation period, the monthly rate of CT-Scans was constant (p for trend = 0.267) at 291.9 (from 95%CI 240.5 to 343.4) per 1,000 registered patients. The first case of COVID-19 coincided with an abrupt increase by 211.8 (from 95%CI 102.9 to 320.7) per 1,000 patients. Thereafter, the trend of CT-Scans did not change (p=0.576) compared to the pre-pandemic period. The rate of MRI services was 363.5 per 1,000 per registered patients per month (P = <0.0001) with a slightly decreasing trend (coefficient=-5; 95%CI, -6.9 to -3.1).

**Conclusion:**

The findings of this study showed that crises such as COVID-19 can affect the service delivery process. Health policymakers and decision makers should work to prevent potential reductions in health care during events such as COVID-19.

## Introduction

The outbreak of COVID-19 has become a really demanding challenge for health systems worldwide and various countries have implemented different policies to prevent and control this disease (1). In many health centers during the fight against COVID-19, in order to control and prevent the death of patients with serious conditions, the healthcare delivery has been more focused on activities related to COVID-19 and other services have been, partially or totally, reduced, repurposed or even cancelled (2). In order to maintain safety and reduce exposure to COVID-19, health care providers have implemented policies to reduce unnecessary services (3).

In February 2020, the Ministry of Health and Medical Education (MOHME) in Iran announced the first case of COVID-19 (4). With the increasing prevalence of this disease in Iran, many people were hospitalized to prevent death and morbidities caused by COVID-19. Early in the onset of the disease, very difficult conditions occurred for service providers (5).

After the first cases of COVID-19 in Iran, the MOHME began to implement various programs and policies (6). By order of the President, a national committee was formed, dealing with all of the activities related to the pandemic (7). All primary health care (PHC) centers and hospitals were prepared to prevent and control COVID-19 (8). Social and economic constraints were put on the agenda to reduce the disease transmission chain. Schools, universities, sports clubs, local and international travel were restricted (6). The capacity of COVID-19 diagnostic laboratories was quickly expanded to better and quicker identify individuals infected with COVID-19 (9) (7). At the national level, about 4.6 million people have been infected with COVID-19 and about 102,000 have died (10).

The COVID-19 pandemic has affected all health-related activities. The disease has reduced some non-emergency activities of health centers, and in the meantime, many problems have arisen, including how to provide basic services, such as diagnostic imaging, with such a high volume of workload generated by the virus (11). Structural and managerial changes have been implemented to cope with this unprecedented situation (4).

**Lorestan Province**: The province of Lorestan, with an area of about 28,559 square kilometers, is located in the western part of Iran. This province has 11 cities, with a population of about 1,800 thousand people and is one of the most populous provinces in Iran. All cities in the province have public hospitals and Lorestan University of Medical Sciences is the most important provider of health services. Implementation of programs and policies related to health is the responsibility of the university. About 150,000 people have been infected since the beginning of the COVID-19 pandemic, and about 2,100 people have died (12).

Considering the conditions caused by COVID-19 and its impact on health services, we seek to assess the impact of this disease on the number of computed tomography scan (CT-Scans) and magnetic resonance imaging (MRI) services in hospitals in Lorestan province in western Iran.

## Materials and Methods

**Data collection**: Primary health services are provided in primary health centers networks (PHCN), in emergency and inpatient services in 18 public hospitals and most health services are provided in the public sector. Lorestan University of Medical Sciences is the main coordinator of health services in the province. We collected CT-Scans and MRI services data from 18 public hospitals via Vice Chancellor Office, Lorestan University of Medical Sciences from January 2017 to February 2021. The number of CT-Scans and MRI services data were extracted monthly.

**Statistical analysis**: Interrupted time series analysis (ITSA) was conducted to assess the impact of COVID-19 on CT-Scans and MRI services. ITSA was performed using ordinary least squares regression with the number of CT-Scans and MRI services per 1,000 registered persons per month as dependent variable. We also adjusted for seasonality by including each calendar month as an independent variable in the model. We set the ‘intervention’ time as February 2020, the month of the first case of the COVID-19. R software Version 5.3.2 was used for all data analyses and p-values < 0.05 were considered as statistically significant.

**Ethics approval and consent to participate**: The ethics committee of Lorestan University of Medical Sciences (LUMS) approved this study (code no. IR.LUMS. REC.1399.083).

## Results

**Figure 1:**
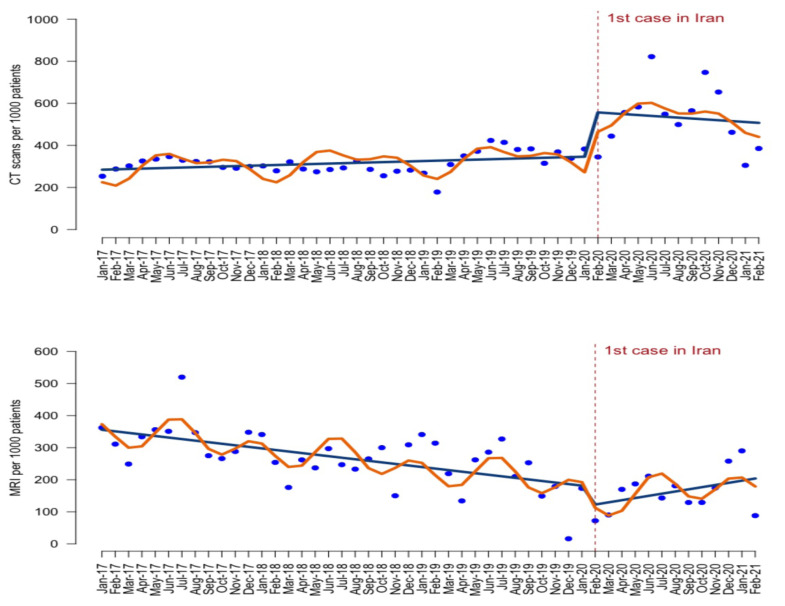
CT-Scans (top) and MRI services (bottom) per 1,000 patients across Iran from January 2017 to February 2021. Wavy line shows the predicted trend based on the seasonally adjusted regression model; Straight line shows the deseasonalized trend

ITSA results are presented in both tabular (Table 1) and graph formats (Figure 1). At the beginning of the observation period, the monthly rate of CT-Scans was constant (p for trend = 0.267) at 291.9 (from 95% CI 240.5 to 343.4) per 1,000 registered patients. The first case of COVID-19 coincided with an abrupt increase by 211.8 (from 95%CI 102.9 to 320.7) per 1.000 patients. Thereafter, the trend of CT-Scans did not change (p=0.576) compared to the pre-pandemic period. The rate of MRI services was 363.5 per 1,000 per registered patients per month (*P* <0.0001) with a slightly decreasing trend (coefficient=-5; 95%CI, -6.9 to -3.1). There was no change when the first case of COVID-19 was declared (*P* = 0.249), but there was an increasing monthly trend of 10.5 (95%CI 0.6 to 20.5; *P* = 0.038) per 1,000 registered patients.

**Table 1:** Change in the number of CT-Scans and MRI services per 1,000 registered patients in Iran according to the seasonal adjusted segmented regression models considering as intervention the first case of COVID-19 in February 2020

Parameter	Coefficients	95% CI	*P* Value
**CT-Scans per 1,000 patients**			
Intercept	291.9	240.5 to 343.4	<0.0001
Baseline trend	1.3	-1.1 to 3.7	0.267
Level change after the intervention	211.8	102.9 to 320.7	<0.0001
Trend change after the intervention	-3.4	-15.4 to 8.7	0.576
**MRI services per 1,000 patients**			
Intercept	363.5	320.9 to 406.2	<.0001
Baseline trend	-5	-6.9 to -3.1	<.0001
Level change after the intervention	-52.2	-142.5 to 38.1	0.249
Trend change after the intervention	10.5	0.6 to 20.5	0.038

## Discussion

The findings of this study showed that CT-Scans services in public hospitals increased abruptly and substantially after the first COVID-19 case. About a year after the onset of COVID-19, the nature and mechanisms of the disease have not yet been fully elucidated. Physicians tried to diagnose patients quickly to prevent serious complications of COVID-19. One of the reasons for this increase could be the increasing use of imaging services by physicians for a faster, better diagnosis and identification of infected people. Studies showed that the use of CT-Scans is of paramount importance in the diagnosis of COVID-19 in health centers and performs well to identify these patients and stratify them according to clinical findings (13). Various methods such as real-time polymerase chain reaction (RT-PCR), antibody and antigen detection methods, and the use of imaging in the health system are used to diagnose COVID-19. The cost of tests such as PCR and serology tests is rather high and CT-Scans imaging findings are less expensive (14). In Iran, RT-PCR and serology tests were particularly expensive and limited. Of course, over time, Iranian companies were able to design and build the required kits, and the capacity to perform diagnostics using these tests increased and probably this is the reason why the CT-Scans stabilized.

Due to the fact that Iranian hospitals have been strictly responsible for hospitalizing and monitoring patients' processes, infections related to COVID-19 patient care have become a concern among service providers and other stakeholders in the community (7). In order to maintain safety and reduce exposure to COVID-19, hospitals have implemented policies to reduce the provision of unnecessary services (6). Meanwhile, many people could not receive these services due to social distancing and fear of being infected in the hospital environment (3).

Our findings showed that the first case of COVID-19 did not affect the use of MRI services. There was a declining trend in MRI services before the era of COVID-19. In Iran, patients visit private offices and public clinics, if necessary, to use MRI services. Many offices and clinics were closed after the outbreak of COVID-19 in Iran and the worsening of the disease and the demand for MRI services for patients by doctors decreased (7). Also, many people were reluctant to go to hospitals and have MRIs for fear of infection. In 2014, the Iranian Ministry of Health implemented a health transformation plan (HTP) aimed at reducing out-of-pocket (OOP) payments and increasing access to services in public hospitals (4). The number of people referring to hospitals has increased and due to the lack of manpower, the working hours of service providers have increased. In the years following the implementation of HTP, hospitals suffered from staff shortages and increased patients' burden. With the increasing burden of COVID-19 disease, and the shortage of manpower, caring for COVID-19 patients made things more difficult for the Iranian health system. One of the decisions in public hospitals was to limit unnecessary services. One of the reasons for the decline in MRI services was the use of MRI ward workforce in other wards related to COVID-19 patients. Of course, in public hospitals, all the efforts of the providers were to make these services available. This reduced the demand for MRI services. Following the stabilization of conditions in hospitals and the increase in access to personal protective equipment and isolation devices, the process of use and MRI services increased as well (6).

This study had some limitations. We did not have access to private hospital data regarding the number of CT-Scans and MRI services provided. Patients may have referred to private hospitals for these services. Private hospitals were not crowded due to low admission or non-admission of COVID-19 patients, and many people were willing to receive their services at these hospitals. There may be other factors contributing to the decline or increase in service delivery during COVID-19 that we cannot address.

In conclusion, the findings of this study showed that crises such as COVID-19 can affect the service delivery process. The trend of diagnostic services such as CT-Scans and MRI has changed due to their role in helping physicians better diagnose diseases after the outbreak of COVID-19 in public hospitals. Health policymakers and decision makers should work to prevent potential reductions in health care during events such as COVID-19.
